# Supplementary material for: Identification of Androgen Receptor Metabolic Correlome Reveals the Repression of Ceramide Kinase by Androgens
Source: Cancers (Basel). 2021 Aug 26;13(17):4307. doi: 10.3390/cancers13174307 (PMC8431577; doi:10.3390/cancers13174307)
Supplement: Supplementary file 1 [file cancers-13-04307-s001.zip › Supplementary material with figure and table legends_Edited after Proofreading-new.pdf]

Supplementary Figures and Legends

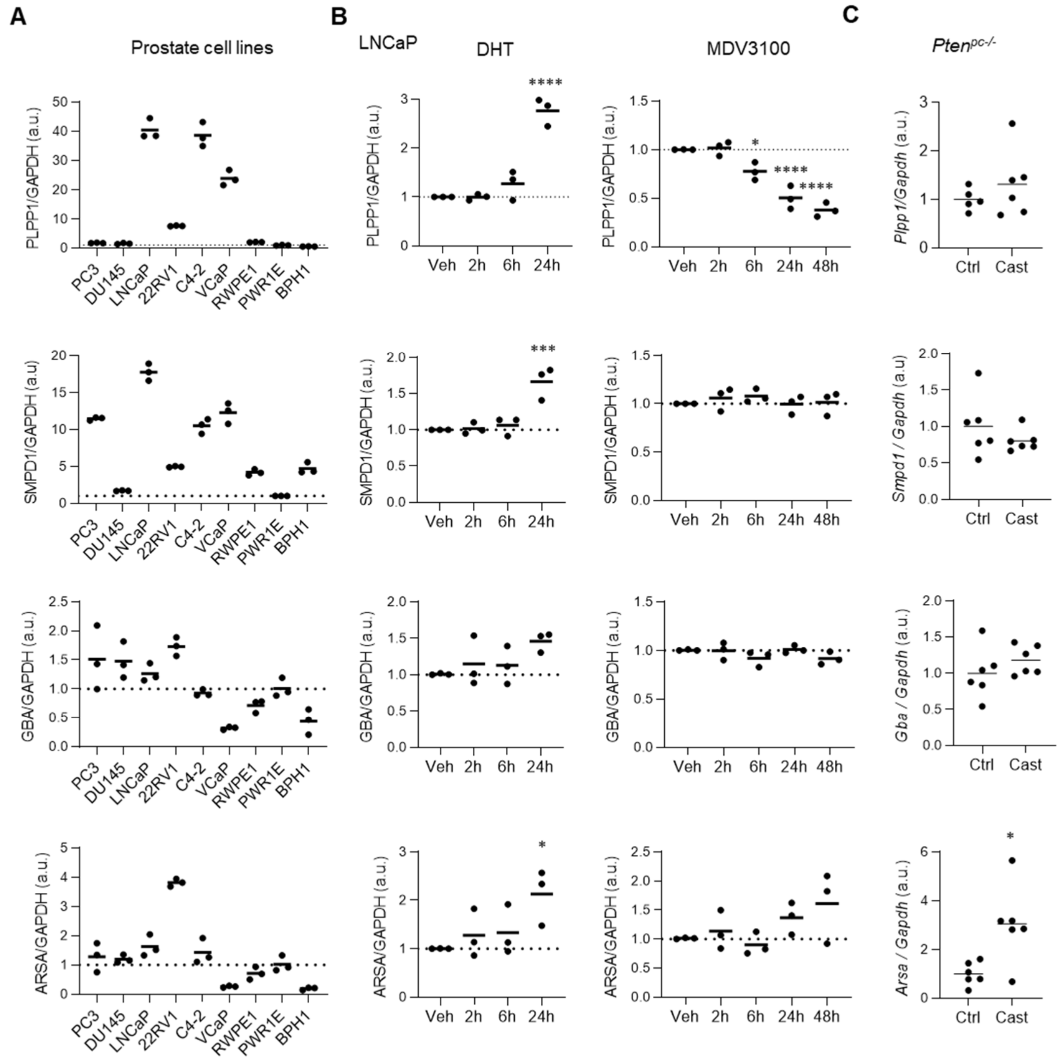

Supplementary Figure S1. Regulation of candidate sphingolipid metabolism enzymes by androgen signaling. (A) Analysis of *PLPP1, SMPD1, GBA* and *ARSA* gene expression by qRT-PCR in a panel of prostate cells. Normalized to the benign cell line PWR1E (n=3 independent experiments). **(B)** Analysis of *PLPP1, SMPD1, GBA* and *ARSA* gene expression by qRT-PCR upon treatment with AR agonist (DHT, 10 nM, left panels) or antagonist (MDV-3100, 10 µM, right panels) in LNCaP cells (n=3 independent experiments). Comparisons made versus vehicle. Dunnett´s multiple comparisons test. **(C)** Analysis of *Plpp1, Smpd1, Gba* and *Arsa* gene expression by qRT-PCR in 6 month-old prostate-conditional *Pten* knock out mice (Pten$^{pc-/-}$, n=6 mice per group) 6 days after performing orchiectomy (Cast) compared to control (Ctrl). One-tailed Mann Whitney test. *p<0.05; ***p<0.001; ****p<0.0001.

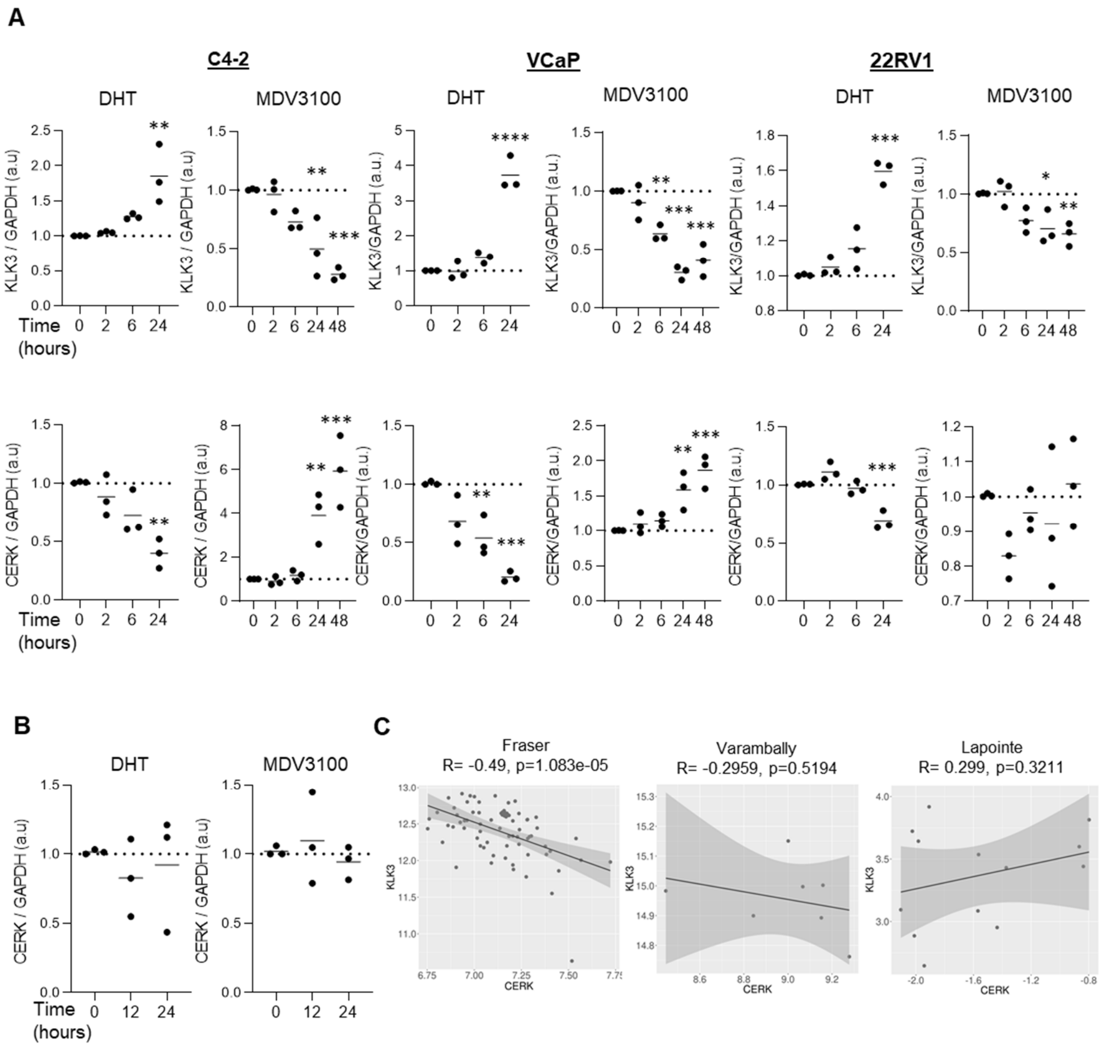

Supplementary Figure S2. Regulation of CERK by androgen signaling in cell lines and patient specimens. (A) Analysis of *KLK3* and *CERK* gene expression by qRT-PCR upon treatment with AR agonist (DHT, 10 nM, left panels) or antagonist (MDV-3100, 10 µM, right panels) in C4-2, VCaP and 22RV1 cells (n=3 independent experiments). Data were normalized to GAPDH expression. Comparisons made versus vehicle. Dunnett´s multiple comparisons test. **(B)** Analysis of *CERK* gene expression by qRT-PCR upon treatment with AR agonist (DHT, 10 nM, left panels) or antagonist (MDV-3100, 10 µM, right panels) in PC3 cells (n=3 independent experiments). Data were normalized to GAPDH expression. Comparisons made versus vehicle. Dunnett´s multiple comparisons test. **(C)** Pearson correlation of *CERK* with *KLK3* in the indicated PCa datasets. *$p<0.05$; **$p<0.01$; ***$p<0.001$; ****$p<0.0001$.

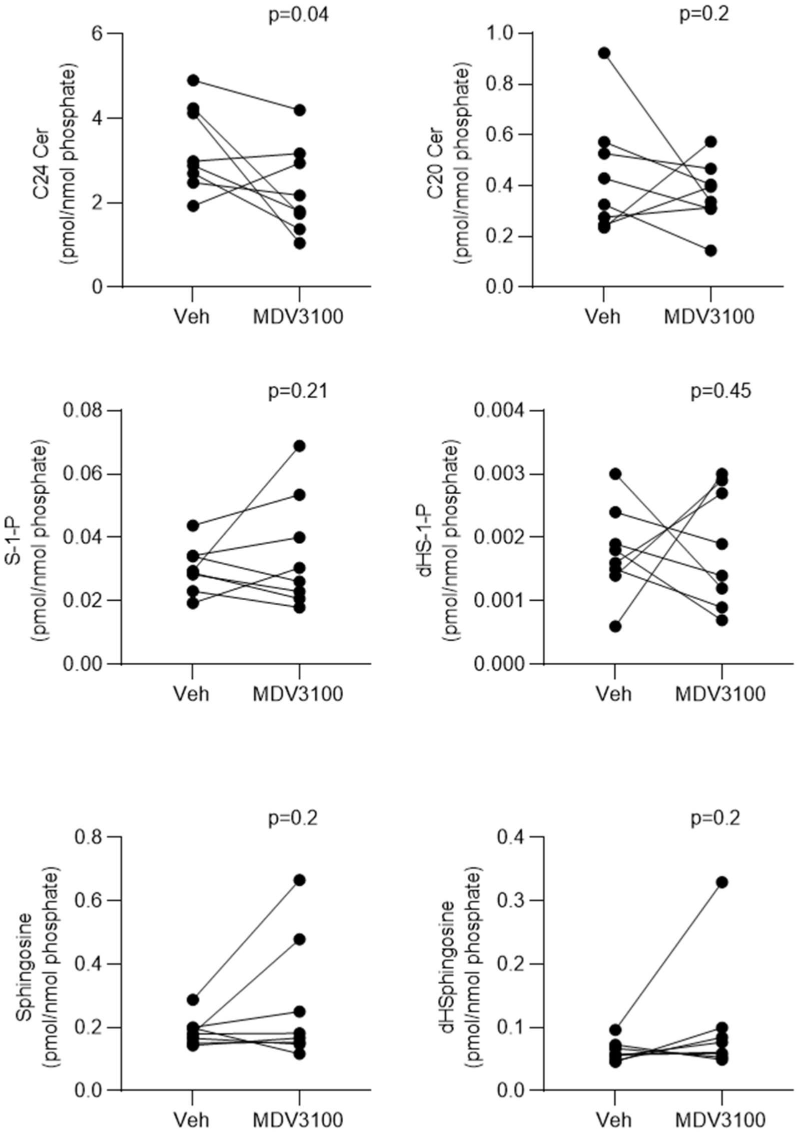

Supplementary Figure S3. LC/MS analysis of phosphorylated and non-phosphorylated ceramides and sphingosine. LC/MS analysis of total C24 and C20 ceramide species (C20 Cer and C24 Cer) as well as phosphorylated and total sphingosine (S-1-P and Sphingosine) and dihydrosphingosine (dHS-1-P and dHSphingosine) upon treatment with AR antagonist MDV-3100 (10 μM, 24 hours) compared to vehicle (Veh). Paired t-test analysis was performed.

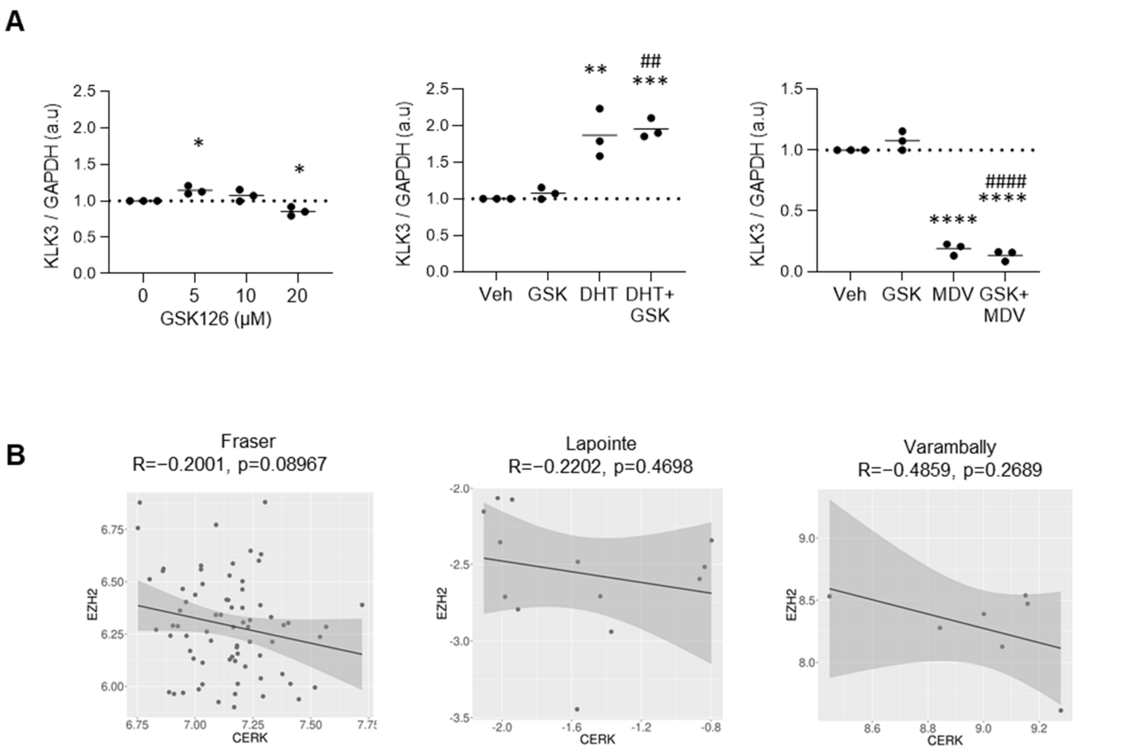

Supplementary Figure S4. Regulation of CERK by EZH2. (A) Analysis of *KLK3* gene expression by qRT-PCR upon treatment with EZH2 inhibitor GSK126 at increasing concentrations (left), in combination with DHT (10 nM, middle) and MDV-3100 (10 µM, right) in LNCaP cells (n=3 independent experiments). ANOVA with Dunnett´s (left) or Tukey´s (center and right) multiple comparison test. Asterisk refer to comparisons against vehicle, hash refers to comparisons against GSK and dollar refers to comparisons against DHT or MDV. **(B)** Pearson correlation of CERK with EZH2 in the indicated PCa datasets. *$p<0.05$; **$p<0.01$; ***$p<0.001$; ****$p<0.0001$; $^{\#\#}$ $p<0.01$; $^{\#\#\#\#}$ $p<0.0001$.

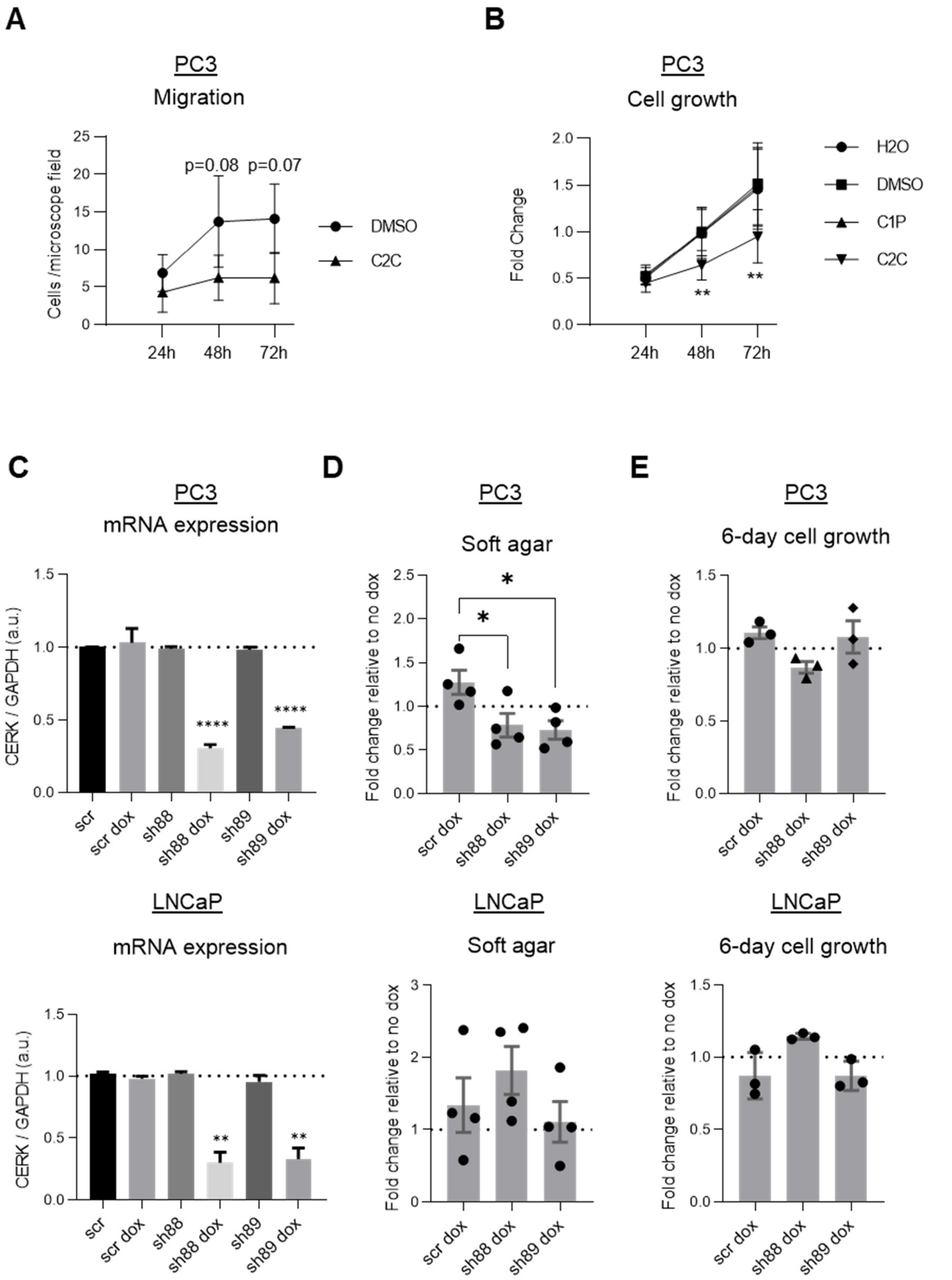

Supplementary Figure S5. Biological consequences of genetic and pharmacological manipulation of ceramide-1-phosphate levels. (A) Quantification of cell migration upon treatment with C2-Ceramide (C2C, 20 μM) in PC3 cells (n=3 independent experiments). Paired t-test.

Comparisons made versus the corresponding vehicle. **(B)** Quantification of cell growth upon treatment with Ceramide-1-phosphate (C1P) and C2C in PC3 cells (n=3 independent experiments). Paired t-test. Comparisons made versus the corresponding vehicle. **(C)** Analysis of CERK gene expression by qRT-PCR upon CERK silencing with two shRNAs (sh88 and sh89) in PC3 (above) and LNCaP (below) cells. Data were normalized to GAPDH expression and compared to no doxycycline (dox). **(D, E)** Effect of CERK silencing on anchorage-independent growth **(D)** and on proliferation after 6 days **(E)** compared to no dox in PC3 (above) and LNCaP (below) cells. n=3 independent experiments. Paired t-tests were performed comparing Dox-induced CERK shRNA vs. Dox-induced scramble shRNA (scr). *p<0.05; **p<0.01; ****p<0.0001.

Supplementary Table S1. Androgen receptor signature genes. The positive or negative transcriptional activation by AR was ascertained in the set of 34 genes identified as consistently regulated by androgens in 6 different experimental studies in the meta-analysis by Jin *et al*. [15] according to the Cancertool interface [28] and the work by Massie *et al*. [27].

Supplementary Table S2. Androgen Receptor metabolic correlome. The AR signature (Supplementary Table 1) was correlated with the metabolic genes (identified as metabolic enzymes and transporters according to KEGG [30] and metabolic co-regulators [31,32]) in 8 different PCa transcriptomics studies containing primary tumor specimens using the Cancertool interface [28]. The genes that exhibited a consistent correlation (correlation coefficient > 0.2 for direct correlations and < -0.2 for inverse correlations) in the majority (>50%) of datasets with available data for a given gene [33-40] were selected.

Supplementary Table S3. Universal Probe Library (Roche) probes/primers and ChIP qPCR primers employed in this study.
